# Self-Modifying Nanointerface Driving Ultrahigh Bidirectional Thermal Conductivity Boron Nitride-Based Composite Flexible Films

**DOI:** 10.1007/s40820-022-00972-9

**Published:** 2022-11-28

**Authors:** Taoqing Huang, Xinyu Zhang, Tian Wang, Honggang Zhang, Yongwei Li, Hua Bao, Min Chen, Limin Wu

**Affiliations:** 1 Department of Materials Science and State Key Laboratory of Molecular Engineering of Polymers, Fudan University, Shanghai, 200433 People’s Republic of China; 2 Department of Physics, University of Michigan–Shanghai Jiao Tong University Joint Institute, Shanghai Jiao Tong University, Shanghai, 200240 People’s Republic of China; 3 Department of Chemistry, Fudan University, Shanghai, 200433 People’s Republic of China

**Keywords:** Thermal management materials, Boron nitride, Thermal conductivity, Interfacial thermal resistance

## Abstract

While boron nitride (BN) is widely recognized as the most promising thermally conductive filler for rapidly developing high-power electronic devices due to its excellent thermal conductivity and dielectric properties, a great challenge is the poor vertical thermal conductivity when embedded in composites owing to the poor interfacial interaction causing severe phonon scattering. Here, we report a novel surface modification strategy called the “self-modified nanointerface” using BN nanocrystals (BNNCs) to efficiently link the interface between BN and the polymer matrix. Combining with ice-press assembly method, an only 25 wt% BN-embedded composite film can not only possess an in-plane thermal conductivity of 20.3 W m^−1^ K^−1^ but also, more importantly, achieve a through-plane thermal conductivity as high as 21.3 W m^−1^ K^−1^, which is more than twice the reported maximum due to the ideal phonon spectrum matching between BNNCs and BN fillers, the strong interaction between the self-modified fillers and polymer matrix, as well as ladder-structured BN skeleton. The excellent thermal conductivity has been verified by theoretical calculations and the heat dissipation of a CPU. This study provides an innovative design principle to tailor composite interfaces and opens up a new path to develop high-performance composites.

**Supplementary Information:**

The online version contains supplementary material available at 10.1007/s40820-022-00972-9.

## Highlights

- The flexible composite film presents ultrahigh thermal conductivity and good thermal management performance in electronic devices.
- An original “self-modified nanointerface” strategy is used to reduce the interfacial thermal resistance between boron nitride and the polymer matrix.
- The ideal phonon spectrum matching between boron nitride nanocrystals and fillers as well as the strong interaction between self-modified fillers and the polymer matrix are the two major contributors to decrease the interfacial thermal resistance.

**Supplementary Information:**

The online version contains supplementary material available at 10.1007/s40820-022-00972-9.

## Graphical abstract

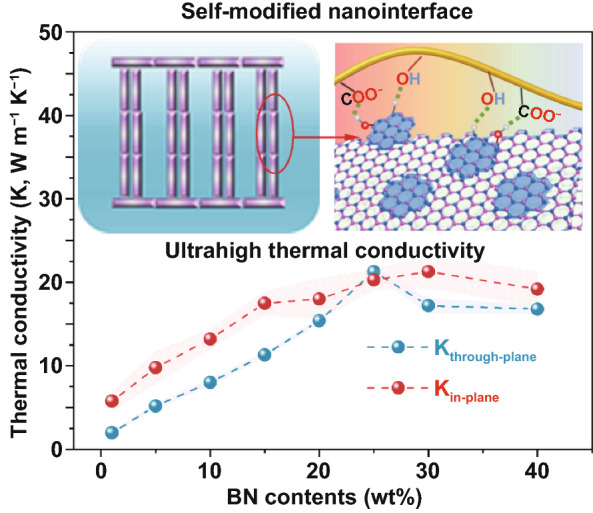

## Introduction

With the rapid development of high-power and high-frequency electronic devices, thermal interface materials (TIMs) applied between heat sources and heat sinks have become vital components to remove excess heat from electronic systems [1]. Given the easy processing, low density and good flexibility properties of polymers, state-of-the-art TIMs are made up of polymer-based composites with high thermal conductivity fillers, such as metal, carbon and ceramic materials [2]. In particular, boron nitride (BN) has attracted tremendous attention during the past decades because of its excellent intrinsic thermal conductivity and electronic insulation, which are indispensable for electronic devices [3–8]. Many interesting methods, such as vacuum filtration and electrospinning, have been explored to develop high thermal conductivity BN-based polymer composite films [9, 10]. However, while all these studies are efficient in improving the in-plane thermal conductivity of composites, their through-plane thermal conductivity is quite low (< 10 W m^−1^ K^−1^) because of the inherent difficulty of two-dimensional (2D) materials to vertically align in composites and the inevitable phonon scattering at the interfaces between fillers and the matrix [11, 12].

To improve the interfacial interactions between metallic or ceramic fillers and polymer matrices, organic molecule-based surface modification is considered a well-known mainstream strategy [13–15]. However, BN is chemically inert and very difficult to be chemically or physically functionalized by organic coupling molecules due to its high bond energy. The limited chemical grafting of functional groups occurs mostly at the edge or defect sites of BN surfaces, which also causes cleavage of B–N bonds [16], thus decreasing the intrinsic properties of BN. Theoretically, to achieve good interface contact, the interfacial modifiers should have similar chemical structures to the fillers and matrices, thus possessing similar phonon spectral features based on the well-known structure–property relationship between bond types and phonon spectra [17, 18].

To address this issue, in this study, we propose an innovative surface modification strategy called the “self-modified nanointerface (SMN)” here using boron nitride nanocrystals (BNNCs) to reduce the interfacial thermal resistance between BN and the polymer matrix. On the one hand, BNNCs present the same lattice structure as 2D BN fillers, ensuring similar phonon spectral features; on the other hand, BNNCs, which are different from inert BN, have abundant hydroxyl and amino groups to interact with the polymer matrix, thus significantly enhancing interfacial thermal transport. Accordingly, the 25 wt% BN-based flexible polymer composite can achieve a through-plane thermal conductivity as high as 21.3 W m^−1^ K^−1^, which is more than twice the reported maximum, while maintaining a high in-plane thermal conductivity of 20.3 W m^−1^ K^−1^. The molecular dynamics simulation indicates that the ideal phonon spectrum matching between BNNCs and BN fillers and the strong interaction between self-modified fillers and the polymer matrix are the two major contributors to decreasing the interfacial thermal resistance.

## Experimental and Calculations

### Experimental of Composite Film

#### Synthesis of BNNCs

Functionalized BNNCs were synthesized by a simple hydrothermal route. Typically, 0.1 g boric acid powder was dissolved in distilled water (10 mL), followed by the addition of 0.035 g melamine. The mixture was stirred at 90 ℃ until fully dissolved and hydrothermally heated in a Teflon-equipped stainless-steel autoclave at 200 ℃ for 16 h. After the reaction, the solution was dialyzed for 2 days and freeze-dried to obtain BNNC powder.

#### SMN for Preparation of the Polymer/s-BN Dispersion

Commercial h-BN powder (1 g) was dispersed in 100 mL 2-propanoal with 0.02 g BNNCs and stirred for more than 24 h at room temperature, followed by centrifugation at 12,000 rpm for 10 min. The s-BN was collected and dried with a freeze-drying process. Certain weights of sodium alginate (SA) and polyvinyl alcohol (PVA) were dissolved in water with vigorously stirring at 90 ℃ for 30 min, followed by stirring with 0.05 wt% citric acid at room temperature for 24 h. After stewing at room temperature for 24 h, a certain amount of s-BN powder was mixed under gentle stirring for another 30 min to obtain a gel. The mixed slurry can also be properly evacuated by a vacuum pump to further eliminate air bubbles generated by stirring.

#### Fabrication of Polymer/BN Composite Films

The above gel was dripped on a flat substrate and visible air bubbles were removed carefully. Monolith ice containing Ca^2+^ ions (3 wt% CaCl_2_) was pressed on the gel to form a flat film. After 10 min, the monolith ice was removed, and the frozen gel film on the substrate was submerged into ethanol for 30 min, followed by solvent exchange in acetone. The composite film was obtained after drying at 60 ℃ and low humidity (below 15%).

### Theoretical Analysis

The interfacial thermal resistance was calculated by non-equilibrium molecular dynamics (NEMD) implemented with the large-scale atomic/molecular massively parallel simulator (LAMMPS) package [19, 20]. The interatomic interactions in the polymeric material were described by the polymer consistent force field (PCFF) [21], while the bonds inside the graphene and h-BN layer were described by the parametrized Tersoff potential for hybrid nanostructures [22]. For the interactions between the graphene layers, h-BN layers and polymer, a universal force field (UFF) [23] was employed. Moreover, the hydrogen bonds formed at the PVA/h-BN and PVA/graphene interfaces were described by the Dreiding force field [24].

In NEMD simulations, a heat bath and a heat sink were maintained on two sides of the simulation domain to generate a steady heat flux across the domain. A temperature drop appears at the interfaces, and the interfacial thermal resistance ${R}_{I}$ can be obtained by

1 $$R_{I} = \frac{{{{\Delta }}T}}{{Q/A}}~$$

where $\Delta T$ is the temperature gap at the interface, $Q$ is the heat flux and *A* is the area of the interface. The heat flux direction is parallel to the longest dimension of the simulation boxes. For the PVA/h-BN, PVA/graphite/h-BN and PVA/h-BN systems, simulation boxes were built with sizes of 17 × 17 × 103, 17 × 17 × 107 and 17 × 17 × 107 Å^3^, respectively. Periodic boundary conditions were applied in all three directions. These systems were first relaxed at 300 K and normal pressure in canonical (NVT) and isothermal–isobaric (NPT) ensembles. After relaxation, the density of the relaxed PVA was 1.3 g cm^−3^, which is consistent with previous experimental results [25]. After the fully relaxed systems were obtained, the atoms at the two ends of the structures were fixed. Meanwhile, two regions with a length of 1 nm and adjoined to the fixed regions were coupled to hot and cold Langevin heat baths. The temperature of the heat source was increased to 350 K, while that of the heat sink was reduced to 250 K. For the NEMD process, the time step was set to 1 fs, and a microcanonical (NVE) ensemble was applied for the whole process. After the systems reached the steady state, the local temperature and heat flux were averaged over the time interval of 1 ns. According to our simulations, the ${R}_{I}$ of PVA/h-BN is 5.18 $\times$ 10^–8^ m^2^ K W^−1^, which agrees with previous experiments [26, 27]. After modification of the interfaces, the ${R}_{I}$ values of PVA/graphite/h-BN and PVA/h-BN decreased to 2.88 $\times$ 10^–8^ and 1.40 $\times$ 10^–8^ m^2^ K W^−1^, respectively.

## Results and Discussion

### Characterization of Polymer/s-BN Composite Films

**Fig. 1:**
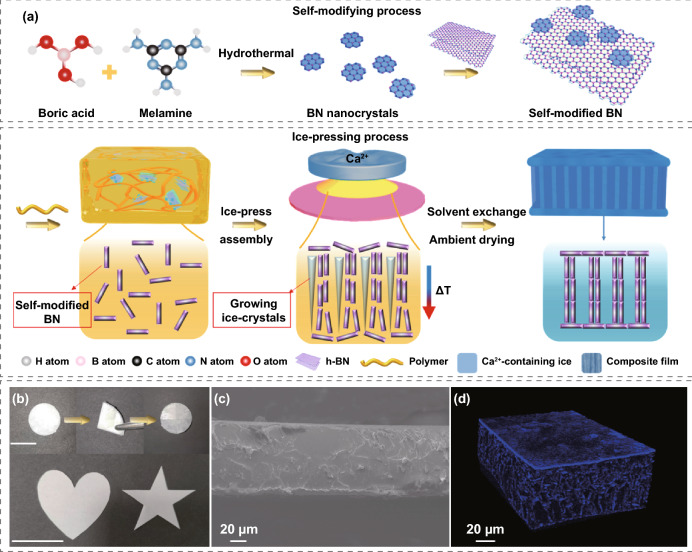
Fabrication and basic characterization of the polymer/s-BN composite film. **a** Schematic illustration presenting the fabrication process of the polymer/s-BN composite film. **b** Photograph, **c** cross-sectional SEM image and **d** nano-CT image of the composite film. The scale bar in **b** is 2 cm. The obtained composite film shows good bendability and ease of cutting

Figure 1a schematically illustrates the SMN strategy to fabricate the BN-based polymer composites. BNNCs were synthesized by a hydrothermal process with melamine and boron acid and separated from the reactant by dialysis. BNNC-based self-modified BN (s-BN) was directly obtained by mixing the commercial BN powder and BNNCs in isopropyl alcohol, during which the BNNCs readily anchored onto the BN surfaces via strong intermolecular interactions [16]. The s-BN was dispersed into an aqueous polymer solution containing SA and PVA to obtain a homogenously mixed gel. This mixed gel was dripped onto a flat surface to fabricate an 80 μm thick composite film by an ice-press assembly strategy as we previously reported (Table S1) [28]. The obtained film can endure multiple folding without any breakage and can be cut into different shapes, indicating excellent flexibility and usability (Fig. 1b). The cross section scanning electron microscopy (SEM) image of the composite film in Figs. 1c and S1 displays a compact morphology without obvious interfacial debonding between the polymer and BN. The nanoscale X-ray computed tomography (nano-CT) image in Fig. 1d clearly presents a lamellar structure at the top and bottom surfaces and vertically connecting BN chain-like fillers inside the film, which provide ideal bidirectional phonon pathways.

**Fig. 2:**
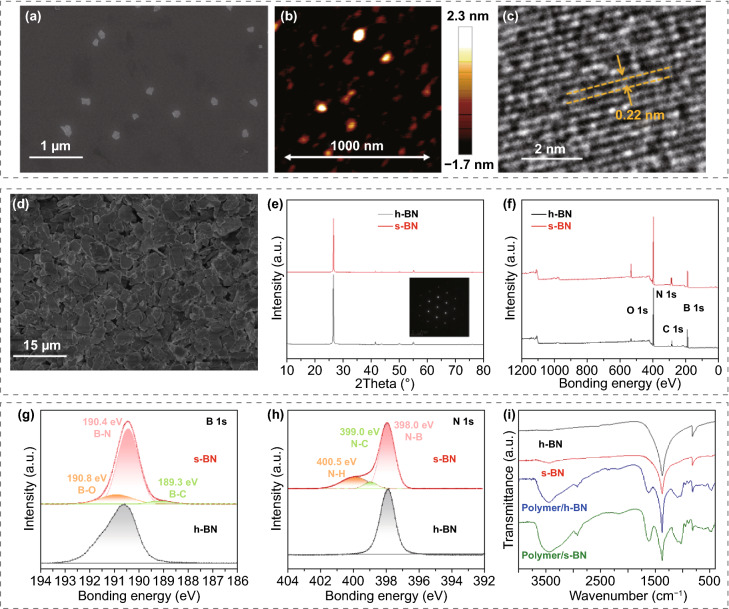
Characterization of BNNCs, s-BN and the polymer/s-BN composite film. **a** SEM, **b** AFM and **c** HRTEM images of BNNCs. **d** SEM image of s-BN with lateral sizes of 10–15 μm. Structural evolution observed via **e** XRD and **f–h** XPS for h-BN and s-BN. The inset in **e** presents the SAED pattern of s-BN with a perfect hexagonal lattice. **i** FTIR spectrum evolution of h-BN, s-BN, polymer/h-BN and polymer/s-BN

SEM and atomic force microscopy (AFM) images of BNNCs reveal a layered structure with a uniform diameter of 100 nm and a thickness of 1–4 nm (Figs. 2a, b and S2). High-resolution transmission electron microscopy (HRTEM) images of BNNCs demonstrate good crystallinity with a clear lattice parameter of 0.22 nm, which corresponds to the (100) lattice fringes of hexagonal BN (h-BN) (Fig. 2c). The representative BNNCs Fourier transform infrared (FTIR) spectrum displays abundant OH/NH groups, as evidenced by the wide peak at 3380 cm^−1^ (Fig. S3). The lateral size of s-BN is approximately 10–15 μm (Fig. 2d), consistent with that of the original h-BN (Fig. S4). Figure 2e–h shows the compositional and structural evolution from h-BN to s-BN revealed by X-ray diffraction (XRD, Fig. 2e) and X-ray photoelectron spectroscopy (XPS, Fig. 2f–h). The XRD and inset selected area electron diffraction (SAED) patterns of s-BN in Fig. 2e can be well indexed to h-BN (JCPDS No. 34–0421), suggesting that almost no obvious defect was created in s-BN. Compared with h-BN, the elemental contents of s-BN are quite different. Specifically, according to pulse heating inert gas fusion-infrared absorption, XPS and thermal gravimetric analysis, the oxygen content increases from 0.1 wt% for h-BN to 0.6 wt% for s-BN (Fig. S5), implying that oxygen comes from the BNNCs anchored on the surface. The high-resolution XPS scans of s-BN present an extra B–O peak at 190.8 eV (B 1* s*) and an extra N–H peak at 400.5 eV (N 1* s*) in Fig. 2g and h, respectively. Therefore, the self-modification strategy can introduce functional groups onto the surfaces of s-BN without causing structural defects.

SA and PVA were chosen as the polymer matrix owing to their good water solubility, low toxicity and good mechanical robustness. More importantly, the abundant oxygen-containing polar groups (–OH, –COO– and = O) of SA and PVA have strong hydrogen-bonding interactions with the –OH and –NH groups of s-BN, which can guarantee a strong interface between the two phases. This interfacial interaction can be further confirmed by FTIR spectra (Fig. 2i), in which h-BN shows two strong characteristic peaks at 1376 and 813 cm^−1^, corresponding to the in-plane stretching of the B–N bond and out-of-plane bending of B–N–B, respectively. After self-modification by BNNCs, a new characteristic broadband peak at approximately 3424 cm^−1^ appears, indicating that a large amount of –OH NH groups have been generated on the s-BN surfaces. A widened broadband at 3440 cm^−1^ can be observed for the polymer/s-BN composite compared with the polymer/h-BN composite, evidencing that intermolecular hydrogen bonds formed between s-BN and the polymer matrix.

### Thermal Performance of Polymer/s-BN Composite Films

**Fig. 3:**
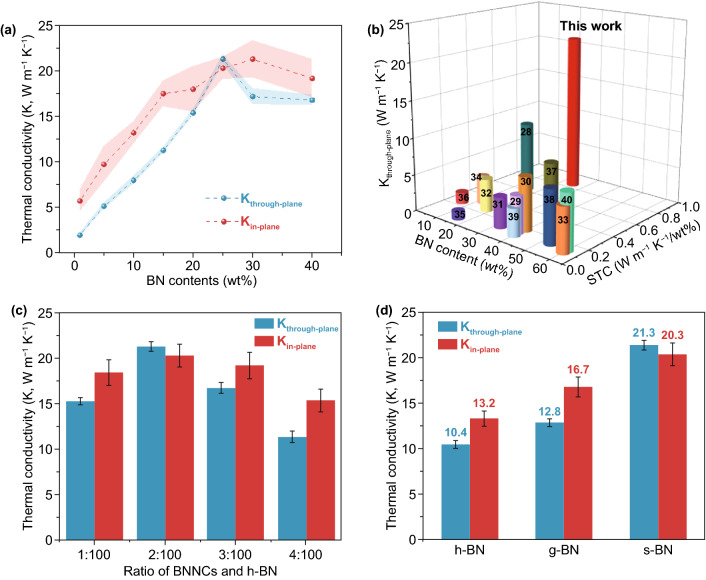
Thermal performance of the polymer/s-BN composite film. **a** Thermal conductivity vs. BN content. **b** Comparison of the through-plane thermal conductivity and specific thermal conductivity (STC, = TC/filler loading (%)) of the polymer/s-BN composite film with those of different composite materials. The polymer/s-BN composite film presents more than twice the reported maximum for BN-based composites. Thermal conductivity with different ratios of BNNCs and h-BN **c** and different fillers **d**

The bidirectional thermal conductivity of the composite film was determined from the thermal diffusivity and specific heat capacity (Fig. S6). Figure 3a shows that both the horizontal and vertical thermal conductivity gradually increases with increasing s-BN content. A point of inflection occurs at approximately 25 wt%, at which the through-plane thermal conductivity reaches the highest value of 21.3 W m^−1^ K^−1^, which is considerably higher than those of previously reported BN-based composites and more than twice the reported maximum (9.8 W m^−1^ K^−1^ at 50 wt% BN, Fig. 3b and Table S2) [28–40], at a simultaneously high in-plane thermal conductivity of 20.3 W m^−1^ K^−1^. The different inflection points of thermal conductivity in the through-plane and in-plane directions stem from the different film volume shrinkage after solvent exchange and ambient drying process, which affect the construction of complete thermal conductive pathway in two directions [41–43]. Further comparison through the specific thermal conductivity (STC = TC/filler loading) demonstrates that our polymer/s-BN composite film presents a value of 0.85, which is also obviously greater than those of the previously reported BN-based composites. All these experimental results and comparisons indicate that the self-modification strategy we present here can use the loaded BN with maximum efficiency.

Since BNNC act as a thermal linker between BN and the polymer, the thermal conductivity of polymer/s-BN composites with different weight ratios of BNNCs to h-BN was examined. As shown in Fig. 3c, too few BNNCs are insufficient to wrap the h-BN plates, while excessive BNNC species wrapping h-BN would bring about low efficiency of heat transfer between h-BN and the polymer. This is because the BNNCs have a lower intrinsic thermal conductivity than h-BN [11], although they have abundant groups to improve interfacial interactions with polymer matrix.

Without any modification, the h-BN-based composite shows vertical and horizontal thermal conductivity of only 10.4 and 13.2 W m^−1^ K^−1^, respectively, as shown in Fig. 3d. Given that graphene oxide has abundant OH groups to interact with the polymer matrix and only a small lattice mismatch (less than 2%) with BN [19], it was also used to wrap BN to fabricate a g-BN-based composite, showing vertical and horizontal thermal conductivity of 12.8 and 16.7 W m^−1^ K^−1^, respectively (Fig. S7), which are still lower than those of the s-BN-based composite. It should be noted that thermal conductivity of BNNCs (4.6 W m^−1^ K^−1^) is slightly lower than that of graphene oxide (7.8 W m^−1^ K^−1^). This suggests that the good lattice matching between BNNCs and BN facilitates interfacial thermal transport and thus an increase in the overall thermal conductivity.

**Fig. 4:**
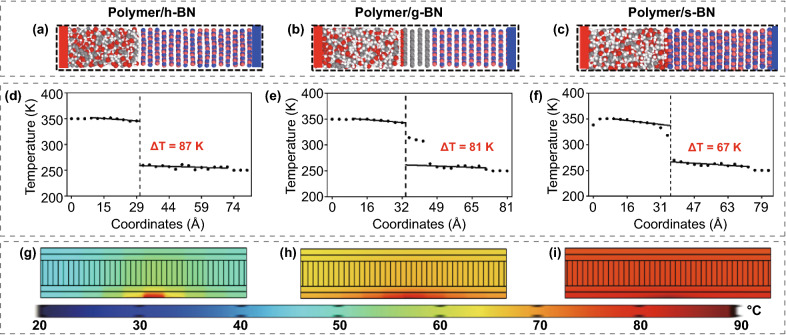
Theoretical analysis of the interfacial thermal resistance between BN and polymers. **a–c** NEMD simulation models and **d–f** temperature configurations of the interfaces in polymer/h-BN, polymer/g-BN and polymer/s-BN composite films. **g–i** Finite element numerical simulation of heat conduction processes in polymer/h-BN, polymer/g-BN and polymer/s-BN composite films

Furthermore, we used NEMD simulations to calculate the thermal transport across the interface between BN and the polymer, as shown in Fig. 4a–c, and evaluate the effectiveness of interface optimization with different modifiers. The specific description of the NEMD simulation can be seen in the theoretical analysis part of the following methods section. The temperature vs. model coordinates of the three systems are shown in Fig. 4d–f. The temperature gap (∆*T*) at the polymer/s-BN interface is 67 K, which is lower than those at the polymer/h-BN (87 K) and polymer/g-BN (81 K) interfaces. According to the simulation results, the interfacial thermal resistance (*R*_*1*_) between pristine BN and the polymer is calculated to be 5.18 × 10^–8^ m^2^ K W^−1^, which agrees with previous experiments [26, 27]. After functionalization with graphene oxide, the *R*_*1*_ of this model is reduced to 2.88 × 10^–8^ m^2^ K W^−1^. The abundant –OH groups and similar crystal lattices between graphene and BN contribute to the decrease in the interfacial thermal resistance. When BN is self-modified by BNNCs, the same crystal lattices of BNNCs and BN lead to a further reduction of *R*_*1*_ to 1.40 × 10^–8^ m^2^ K W^−1^. These results confirm that the SMN strategy here can improve the interfacial phonon transport and thus the thermal conductivity of the composite.

Finite element simulations were then carried out to visualize the heat transfer process of the thermal management materials as a function of the BN-polymer interfacial thermal resistance. The corresponding temperature distribution is displayed in Fig. 4g–i, and the parameters are listed in Table S3. The thermal resistances of the model were set as 5.18 × 10^–8^, 2.88 × 10^–8^ and 1.40 × 10^–8^ m^2^ K W^−1^, as calculated above, which agree with the interfacial thermal resistance between the polymer and h-BN, g-BN and s-BN. By applying a localized heat source at the bottom, the heat spreads in the parallel direction and is transported to the top surface over time. The temperature distribution contours were obtained to reflect the thermal transfer process inside the materials. Figure 4g–h demonstrates that the h-BN- and g-BN-based composites only present a weak heat flux distribution and a low top surface temperature due to the very large interfacial thermal resistance of the composites. In contrast, the model composed of s-BN displays the highest top surface temperature (Fig. 4i). Hence, the polymer/s-BN composite film exhibits more efficient heat conduction than its polymer/g-BN counterparts, which again highlights the significance of interface engineering for achieving high thermal conductivity with low filler percolation composites.

### Basic Performance and Thermal Management Capability of the Polymer/s-BN Composite Films

**Fig. 5:**
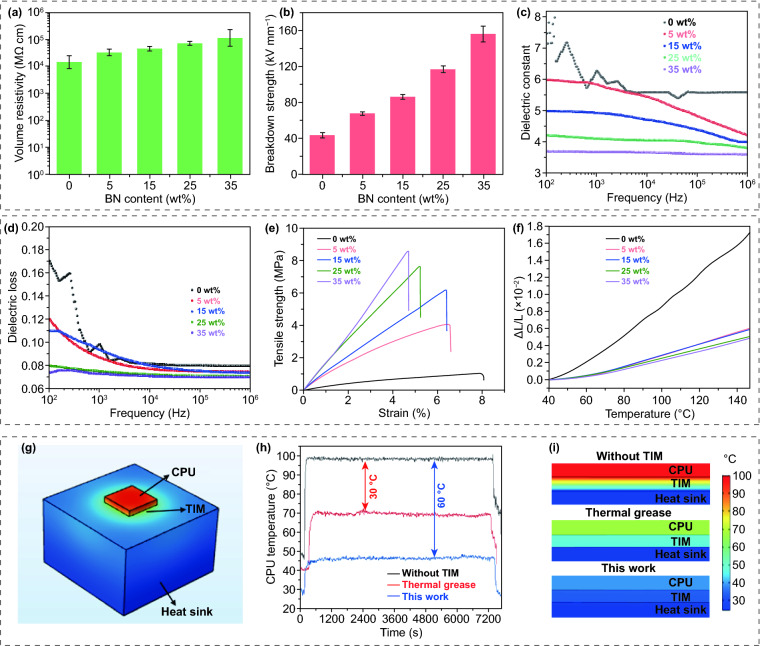
Basic performance and thermal management capability of the polymer/s-BN composite film. **a** Volume resistivity, **b** breakdown strength, **c** dielectric constant, **d** dissipation factor, **e** typical tensile stress curves and **f** dimension changes with temperature for polymer/s-BN composite films with different BN contents. **g** Configuration for TIM performance measurement. **h** Core temperature evolution curves for the CPU with or without TIMs as a function of time. **i** Cross-sectional temperature distribution of the TIM obtained based on the system in **g**. The CPU with our sample shows the lowest temperature since the composite film can effectively transfer heat to the heat sink

Having demonstrated the ultrahigh thermal conductivity performance of the s-BN-based flexible composite film, we further explored its practical application. For microelectronic packaging, to decrease the parasitic capacitances, high electrical resistivity, high breakdown strength, low dielectric constant and low dielectric loss are required for thermal management materials. The composite film with 25 wt% s-BN exhibits a high volume resistivity of over 7 × 10^10^ Ω cm (Fig. 5a), far exceeding the standard for electrical insulation (10^9^ Ω cm), indicating the excellent electrical insulation property to ensure electrical safety. In addition, the breakdown strengths of the composites with different BN contents shown in Fig. 5b demonstrate an incredible increase from 40 to 150 kV mm^−1^. This is because the BN skeleton inside the film can generate a robust scaffold hampering the onset of electromechanical failure. The dielectric constants and dissipation factors were measured as a function of frequency, as shown in Fig. 5c, d. Apparently, the dielectric constant and dissipation factor decrease with the addition of BN. Since the dielectric properties of polymer composites are closely related to the dispersibility, polarity and reactivity of the BN filler, the good dispersion of BN in the matrix and the weak polar structures, such as B–N of BN, contribute to the reduction of the dielectric constant and dielectric loss in the present work. These results illustrate that the composite film can act as an effective insulating shield against space charge conduction and current leakage. Furthermore, with a filler content of 25 wt%, our composite film shows a tensile strength of 7.6 MPa (Fig. 5e). In addition, based on the length thermal expansion ratio (*△L*/*L*) of less than 0.6 × 10^–2^ in Fig. 5f, the extremely low coefficient of thermal expansion of less than 60 ppm K^−1^ can prevent our film from disconnecting from overheated electronic devices, further avoiding thermal failure at high temperature.

Due to the comprehensive properties, including ultrahigh thermal conductivity, good mechanical strength, bendability, excellent electrical insulation and shape stability, the polymer/s-BN composite film provides great potential as a TIM for cooling high-power density devices. Thus, to evaluate the cooling efficiency of this film in real operating conditions, commercially widely used thermal grease and our sample with the same size of 3 × 3 cm^2^ and thickness of 80 μm were separately placed between a CPU (54 W, Fig. S8) and an aluminum heat sink, as shown in Fig. 5g. Figure 5h presents the temperature evolution of the CPU as a function of running time with and without the TIMs operating at maximum power. Compared with the case without TIMs, the steady-state CPU temperature using our composite film can considerably decrease by 60 °C, which is larger than the decrease with thermal grease (30 °C), demonstrating considerably efficient cooling capacity. A computational fluid dynamics simulation based on COMSOL was then carried out to calculate the cooling performance of the two TIMs based on the steady-state CPU temperature shown in Fig. 5h. The detailed calculation process is given in Table S4. As demonstrated in Figs. 5i and S9, the simulated temperature profiles show that the heat generated by CPU operation can effectively be transferred to the heat sink through the polymer/s-BN composite film. As a result, combining ultrahigh thermal conductivity and excellent electrical insulation, our composite film with self-modified BN presents remarkable heat dissipation capability for TIM applications.

## Conclusions

In summary, we have demonstrated an original “SMN” strategy using BNNCs to match 2D BN thermally conductive fillers in a crystal lattice and produce strong hydrogen-bonding interactions with the polymer matrix. This can efficiently decrease the interfacial thermal resistance and considerably enhance the thermal conductivity, achieving an ultrahigh value of 21.3 W m^−1^ K^−1^ for the through-plane thermal conductivity during maintaining excellent in-plane thermal conductivity, which is more than twice the reported maximum. The ultrahigh thermal conductivity and excellent electronic insulation make the as-obtained s-BN-supporting flexible composite be the most promising heat dissipation media for next-generation high-power electronic devices. This design idea of self-modified nanointerface provides an innovative strategy to improve the interfacial interactions and may be extended to the fabrication of other organic–inorganic hybrid and composite materials that are not easily fabricated by traditional organic molecule-based surface modification strategies.

### Supplementary Information

Below is the link to the electronic supplementary material.

Supplementary file1 (PDF 714 KB)

## References

[1] Waldrop MM (2016). The chips are down for Moore’s law. Nature.

[2] Feng CP, Wei F, Sun KY, Wang Y, Lan HB (2022). Emerging flexible thermally conductive films: mechanism, fabrication, application. Nano-Micro Lett..

[3] Q. Cai, D. Scullion, W. Gan, A. Falin, S. Zhang et al., High thermal conductivity of high-quality monolayer boron nitride and its thermal expansion. Sci. Adv. (2019). 10.1126/sciadv.aav0129 10.1126/sciadv.aav0129 PMC6555632 31187056

[4] Shen X, Zheng Q, Kim JK (2021). Rational design of two-dimensional nanofillers for polymer nanocomposites toward multifunctional applications. Prog. Mater. Sci..

[5] Pan D, Yang G, Abo-Dief HM, Dong J, Su F (2022). Vertically aligned silicon carbide nanowires/boron nitride cellulose aerogel networks enhanced thermal conductivity and electromagnetic absorbing of epoxy composites. Nano-Micro Lett..

[6] Wang J, Liu D, Li Q, Chen C, Chen Z (2021). Nacre-bionic nanocomposite membrane for efficient in-plane dissipation heat harvest under high temperature. J. Materiomics.

[7] Wang J, Li Q, Liu D, Chen C, Chen Z (2018). High temperature thermally conductive nanocomposite textile by “green” electrospinning. Nanoscale.

[8] Wang J, Wu Y, Xue Y, Liu D, Wang X (2018). Super-compatible functional boron nitride nanosheets/polymer films with excellent mechanical properties and ultra-high thermal conductivity for thermal management. J. Mater. Chem. C.

[9] Chen J, Huang X, Sun B, Jiang P (2019). Highly thermally conductive yet electrically insulating polymer/boron nitride nanosheets nanocomposite films for improved thermal management capability. ACS Nano.

[10] Wu K, Wang J, Liu D, Lei C, Liu D (2020). Highly thermoconductive, thermostable, and super-flexible film by engineering 1D rigid rod-like aramid nanofiber/2D boron nitride nanosheets. Adv. Mater..

[11] Qian X, Zhou J, Chen G (2021). Phonon-engineered extreme thermal conductivity materials. Nat. Mater..

[12] Giri A, Hopkins PE (2019). A review of experimental and computational advances in thermal boundary conductance and nanoscale thermal transport across solid interfaces. Adv. Funct. Mater..

[13] Losego MD, Grady ME, Sottos NR, Cahill DG, Braun PV (2012). Effects of chemical bonding on heat transport across interfaces. Nat. Mater..

[14] Wang H, Xing W, Chen S, Song C, Dickey MD (2021). Liquid metal composites with enhanced thermal conductivity and stability using molecular thermal linker. Adv. Mater..

[15] Jiang F, Cui S, Rungnim C, Song N, Shi L (2019). Control of a dual-cross-linked boron nitride framework and the optimized design of the thermal conductive network for its thermoresponsive polymeric composites. Chem. Mater..

[16] Liu Z, Dibaji A, Li D, Mateti S, Liu J (2021). Challenges and solutions in surface engineering and assembly of boron nitride nanosheets. Mater. Today.

[17] Sun F, Zhang T, Jobbins MM, Guo Z, Zhang X (2014). Molecular bridge enables anomalous enhancement in thermal transport across hard-soft material interfaces. Adv. Mater..

[18] O'Brien PJ, Shenogin S, Liu J, Chow PK, Laurencin D (2013). Bonding-induced thermal conductance enhancement at inorganic heterointerfaces using nanomolecular monolayers. Nat. Mater..

[19] Yao Y, Sun J, Zeng X, Sun R, Xu JB (2018). Construction of 3D skeleton for polymer composites achieving a high thermal conductivity. Small.

[20] Plimpton S (1995). Fast parallel algorithms for short-range molecular-dynamics. J. Comput. Phys..

[21] Sun H, Mumby SJ, Maple JR, Hagler AT (1994). An ab-initio CFF93 all-atom force-field for polycarbonates. J. Am. Chem. Soc..

[22] Kinaci A, Haskins JB, Sevik C, Cagin T (2012). Thermal conductivity of BN-C nanostructures. Phys. Rev. B.

[23] Rappe AK, Casewit CJ, Colwell KS, Goddard WA, Skiff WM (1992). UFF, a full periodic-table force-field for molecular mechanics and molecular-dynamics simulations. J. Am. Chem. Soc..

[24] Mayo SL, Olafson BD, Goddard WA (1990). DREIDING - a generic force-field for molecular simulations. J. Phys. Chem..

[25] Davidson RL (1980). Handbook of Water-Soluble Gums and Resins.

[26] Lin Z, McNamara A, Liu Y, Moon KS, Wong CP (2014). Exfoliated hexagonal boron nitride-based polymer nanocomposite with enhanced thermal conductivity for electronic encapsulation. Compos. Sci. Technol..

[27] Z. Lin, Y. Liu, S. Raghavan, K.S. Moon, S.K. Sitaraman det al., Magnetic alignment of hexagonal boron nitride platelets in polymer matrix: toward high performance anisotropic polymer composites for electronic encapsulation. ACS Appl. Mater. Interfaces **5**(15), 7633–7640 (2013). 10.1021/am401939z 23815609

[28] Huang T, Yang F, Wang T, Wang J, Li Y (2022). Ladder-structured boron nitride nanosheet skeleton in flexible polymer films for superior thermal conductivity. Appl. Mater. Today.

[29] Lei C, Zhang Y, Liu D, Xu X, Wu K (2021). Highly thermo-conductive yet electrically insulating material with perpendicularly engineered assembly of boron nitride nanosheets. Compos. Sci. Technol..

[30] An L, Gu X, Zhong B, Wang J, Zhang J (2021). Quasi-isotropically thermal conductive, highly transparent, insulating and super-flexible polymer films achieved by cross linked 2D hexagonal boron nitride nanosheets. Small.

[31] Xiao C, Guo Y, Tang Y, Ding J, Zhang X (2020). Epoxy composite with significantly improved thermal conductivity by constructing a vertically aligned three-dimensional network of silicon carbide nanowires/ boron nitride nanosheets. Compos. Part B Eng..

[32] Kashfipour MA, Dent RS, Mehra N, Yang X, Gu J (2019). Directional xylitol crystal propagation in oriented micro-channels of boron nitride aerogel for isotropic heat conduction. Compos. Sci. Technol..

[33] Xu X, Hu R, Chen M, Dong J, Xiao B (2020). 3D boron nitride foam filled epoxy composites with significantly enhanced thermal conductivity by a facial and scalable approach. Chem. Eng. J..

[34] Yao Y, Ye Z, Huang F, Zeng X, Zhang T (2020). Achieving significant thermal conductivity enhancement *via* an ice-templated and sintered BN-SiC skeleton. ACS Appl. Mater. Interfaces.

[35] Hong H, Jung YH, Lee JS, Jeong C, Kim JU (2019). Anisotropic thermal conductive composite by the guided assembly of boron nitride nanosheets for flexible and stretchable electronics. Adv. Funct. Mater..

[36] Wang X, Wu P (2019). 3D vertically aligned BNNS network with long-range continuous channels for achieving a highly thermally conductive composite. ACS Appl. Mater. Interfaces.

[37] Wang J, Liu D, Li Q, Chen C, Chen Z (2019). Lightweight, superelastic yet thermoconductive boron nitride nanocomposite aerogel for thermal energy regulation. ACS Nano.

[38] Xue Y, Zhou X, Zhan T, Jiang B, Guo Q (2018). Densely interconnected porous bn frameworks for multifunctional and isotropically thermoconductive polymer composites. Adv. Funct. Mater..

[39] Li X, Li C, Zhang X, Jiang Y, Xia L (2020). Simultaneously enhanced thermal conductivity and mechanical properties of PP/BN composites via constructing reinforced segregated structure with a trace amount of BN wrapped PP fiber. Chem. Eng. J..

[40] Hu Q, Bai X, Zhang C, Zeng X, Huang Z (2022). Oriented BN/silicone rubber composite thermal interface materials with high out-of-plane thermal conductivity and flexibility. Compos. Part A Appl. Sci. Manuf..

[41] Wang ZG, Lv JC, Zheng ZL, Du JG, Dai K (2021). Highly thermally conductive graphene-based thermal interface materials with a bilayer structure for central processing unit cooling. ACS Appl. Mater. Interfaces.

[42] Wang ZG, Liu W, Liu YH, Ren Y, Li YP (2020). Highly thermal conductive, anisotropically heat-transferred, mechanically flexible composite film by assembly of boron nitride nanosheets for thermal management. Compos. Part B Eng..

[43] Wang ZG, Jin YF, Hong R, Du J, Dai K (2022). Dual-functional thermal management materials for highly thermal conduction and effectively heat generation. Compos. Part B Eng..

