# Supplementary material for: Self-Modifying Nanointerface Driving Ultrahigh Bidirectional Thermal Conductivity Boron Nitride-Based Composite Flexible Films
Source: Nanomicro Lett. 2022 Nov 28;15:2. doi: 10.1007/s40820-022-00972-9 (PMC9705632; doi:10.1007/s40820-022-00972-9)
Supplement: Supplementary file 1 — Supplementary file1 (PDF 714 KB) [file 40820_2022_972_MOESM1_ESM.pdf]

Supporting Information for

Self-Modifying Nanointerface Driving Ultrahigh Bi-Directional Thermal Conductivity Boron Nitride-Based Composite Flexible Films

Taoqing Huang[1], Xinyu Zhang[2], Tian Wang[3], Honggang Zhang[2], Yongwei Li[1], Hua Bao[2], Min Chen[1,*] and Limin Wu[1,*]

[1]Department of Materials Science and State Key Laboratory of Molecular Engineering of Polymers, Fudan University, Shanghai 200433, P. R. China

[2]Department of Physics, University of Michigan－Shanghai Jiao Tong University Joint Institute, Shanghai Jiao Tong University, Shanghai 200240, P. R. China

[3]Department of Chemistry, Fudan University, Shanghai 200433, P. R. China

*Corresponding authors. E-mail: lmw@fudan.edu.cn (L. W.), chenmin@fudan.edu.cn (M. C.)

S1 Materials and Characterizations

Materials: Boric acid (AR grade, 99.9%) and melamine (AR grade, 97.0%) were obtained from Aladdin chemistry Co., Ltd, China. Commercial BN powder with average size in range of 10-15 μm was purchased from Qinghuangdao Eno High Tech Material Development Co., Ltd. PVA (molecular weight: 13000-23000 and 98% hydrolyzed) was from Sigma-Aldrich. SA (98 wt%, viscosity 200±20 mpa·s, 1%, 2 °C), citric acid (AR grade, 99.5%) were supplied from Aladdin chemistry Co., Ltd, China. Calcium chloride (AR Grade, 96.0%), 2-propanoal (IPA, AR Grade, 99.5%), ethanol (AR Grade, 99.7%) and acetone (AR Grade, 99.5%) were obtained from Sinopharm Chemical Reagent Co., Ltd., China. All chemicals were received and used without further purification.

Characterization: The morphologies of BNNC, s-BN and composite film were observed by field emission SEM (FESEM, SEM500, Zeiss), AFM (Multimode 8, Bruker) and high resolution transmission electron microscope (HRTEM, JEM-2100F, JEOL). 3D structure of BN scaffolds was imaged using Nano X-ray Computed Tomography (nano-CT, SkyScan 2211, Bruker). The composition and bonding states of h-BN and s-BN were analyzed by an X-ray photoelectron spectrometer (XPS, K-Alpha, Thermo Scientific) X ray diffraction (XRD, D8 ADVANCE, Bruker) curves were recorded by Cu Kα radiation and a scanning speed of 2 °/min to characterize crystalline structure of h-BN and s-BN. The oxygen contents of h-BN and s-BN were measured by pulse heating inert gas fusion-infra-red absorption method. The FTIR test was measured by a Fourier transform infrared (FT-IR) spectra meter ((FTIR, Nicolet 6700, Thermofisher). The mechanical properties of composite films were measured on a universal material testing machine (UTM, 5567A, Instron). The thermal conductivity (K, W $m^{-1}K^{-1}$) was calculated by multiplying thermal diffusivity (α, mm^2 s^{-1}), density (ρ, g cm^{-1}) and specific heat (C_p, J $g^{-1}K^{-1}$).

$$K=\alpha \times C_p \times \rho \tag{S1}$$

Thermal diffusivity was measured through the laser flash technique (LFA 467 HT Hyperflash, Nanoflash, Netzsch). The thermal conductivity of graphene oxide and BNNCs were measured by hot disk method (Hot Disk TPS2500S). Differential scanning calorimetry (DSC Q200, TA instruments) was used to measure specific heat capacity at heating rate of 10 °C min^{-1}. At least three specimens were tested for each sample to obtain reliable data. Volume resistivity was measured by ZC-36 high resistance meter (Shanghai No. 6 Meter Factory Co., Ltd.). Thermal

expansion coefficient was measured by TMA (TA Q400, TA intruments) at heating rate of 5 $^{o}Cmin^{-1}$ from 40 to 150 ^{o}C. Thermal gravimetric (TG) analyses were evaluated by TGA (TGA 1, Mettler Toledo) at heating rate of 20 ^{o}C min^{-1} from 30 to 800 ^{o}C. The breakdown strength was obtained by a dielectric strength tester (HCDJC-100KV, Huace). The frequency-depended dielectric constant and dielectric loss were measured by a precision impedance analyzer (Agilent 4294A, Melrose) at room temperature with the frequency range of 10^2-10^6 Hz.

S2 Supplementary Figures

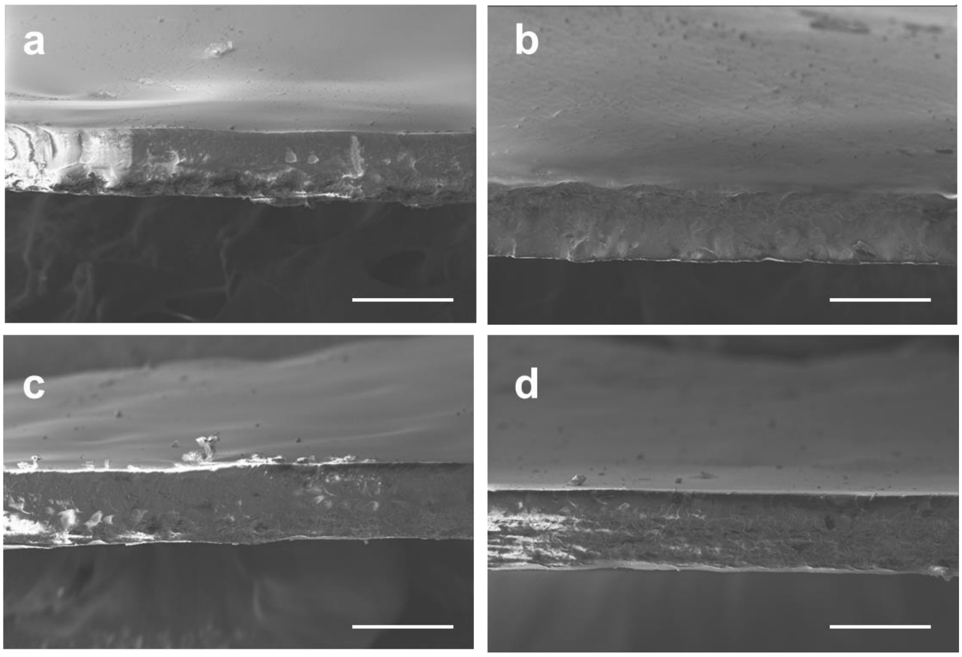

Fig. S1 Cross-sectional SEM images of polymer/s-BN composite film with different filler content. **a** 0 wt%, **b** 5 wt%, **c** 15 wt% and **d** 35 wt%. Scale bar: 100 μm

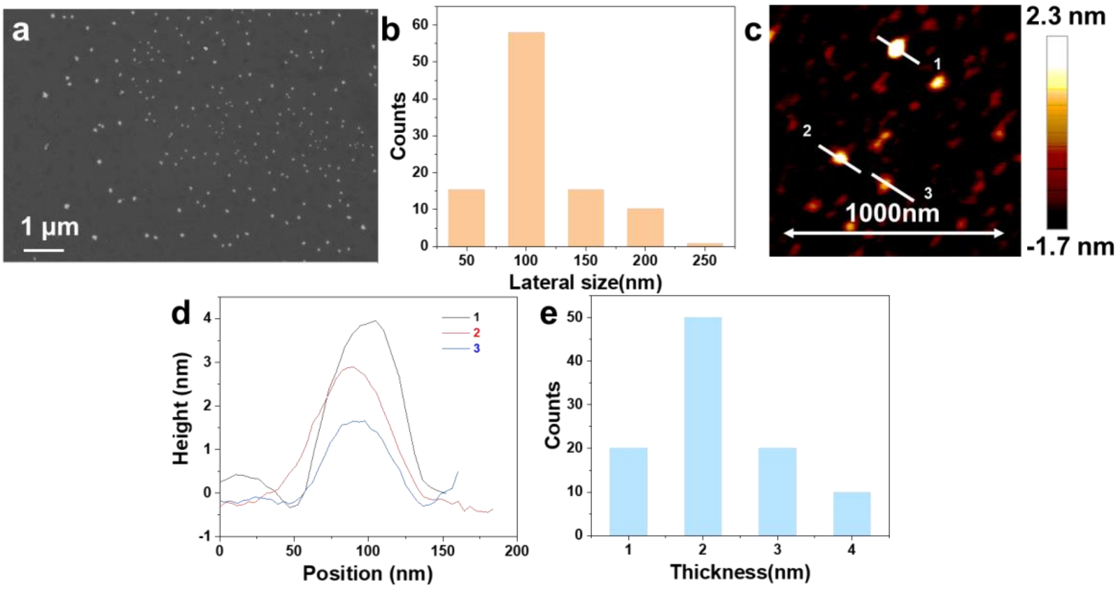

Fig. S2 Characterization of BNNCs. **a** SEM image, **b** lateral size histogram, **c** AFM image, **d** corresponding height variations and **e** thickness histogram of BNNCs

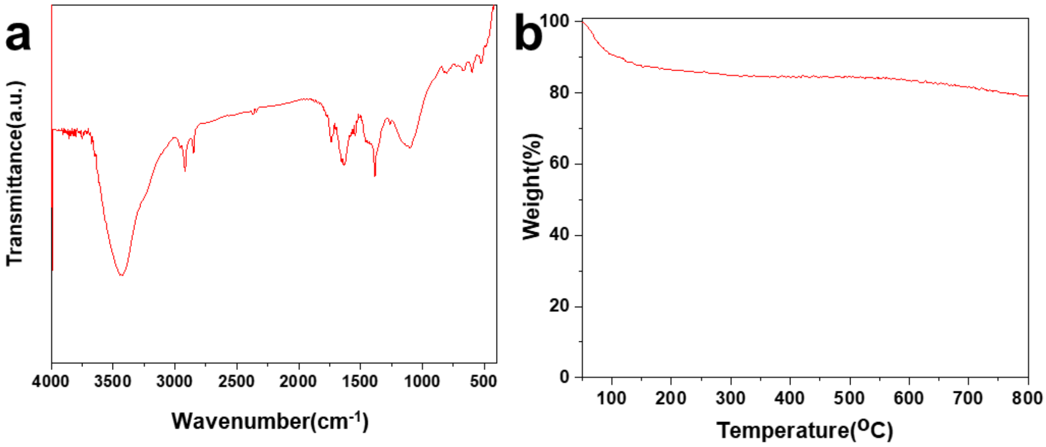

Fig. S3 FTIR transmittance spectrum and TGA curve for the BNNCs

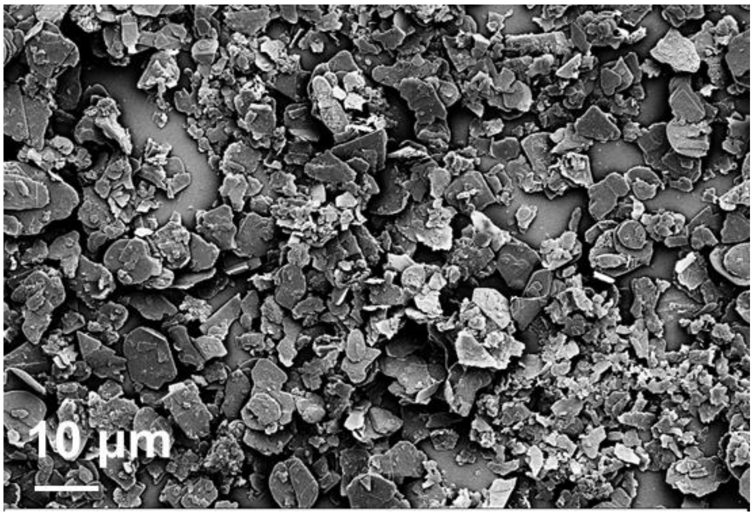

Fig. S4 SEM image of pristine h-BN powder with lateral sizes of 10-15 μm

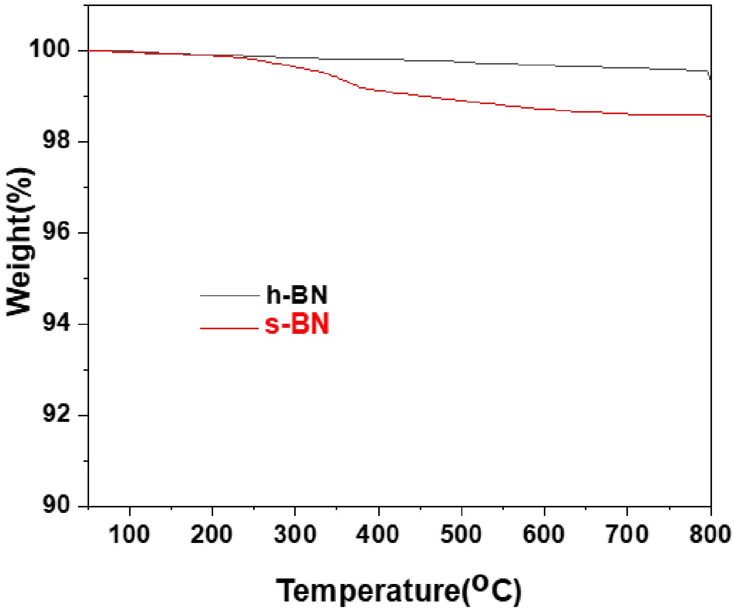

Fig. S5 TGA curves of h-BN and s-BN

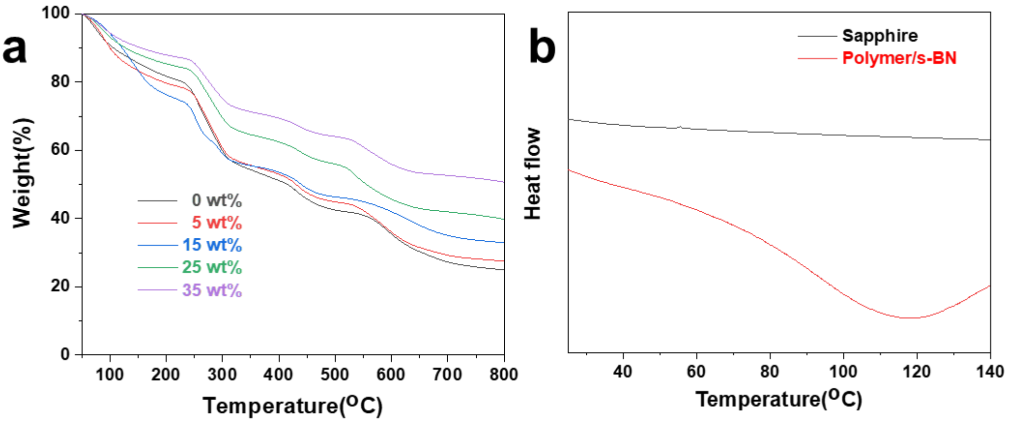

Fig. S6 Thermodynamic analyses of composite films. **a** TGA curves of composite films with different BN contents. **b** DSC curves of composite films (25 wt% BNNS) and Sapphire

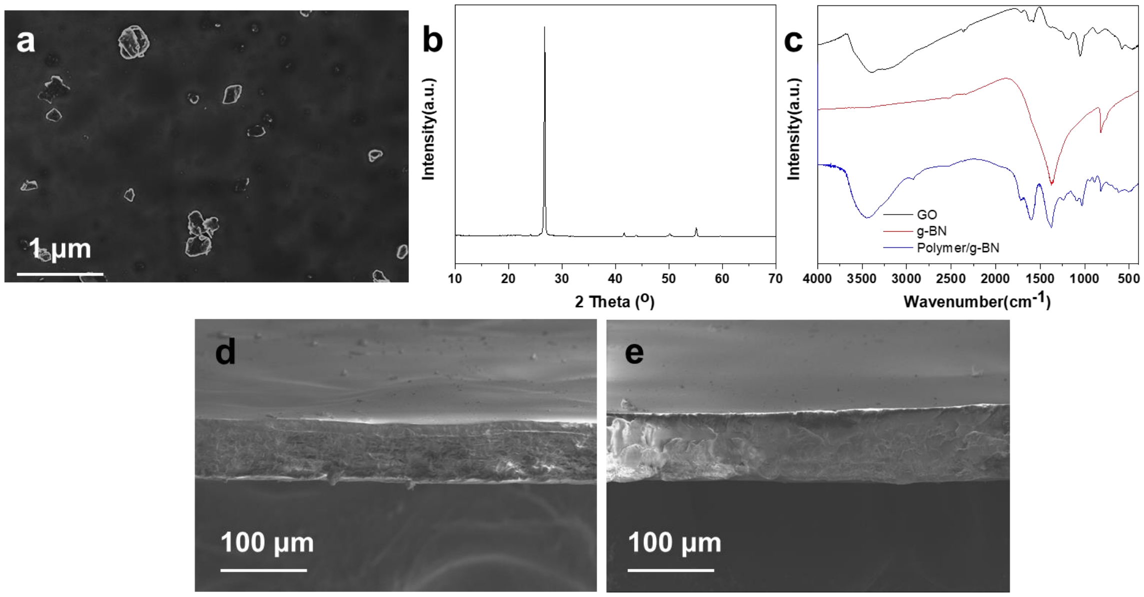

Fig. S7 Basic characterization of the polymer/g-BN composite film. **a** SEM image of graphene oxide nanosheets (GO) with lateral sizes of 100-500 nm. **b** XRD pattern of the g-BN, presenting good crystallinity. **c** FTIR spectrum evolutions of GO, g-BN and polymer/g-BN. Cross-sectional SEM images of polymer/h-BN **d** and polymer/g-BN **e**

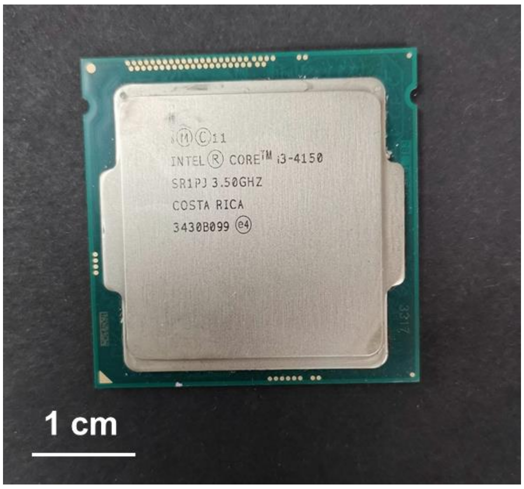

Fig. S8 Digital photo of CPU

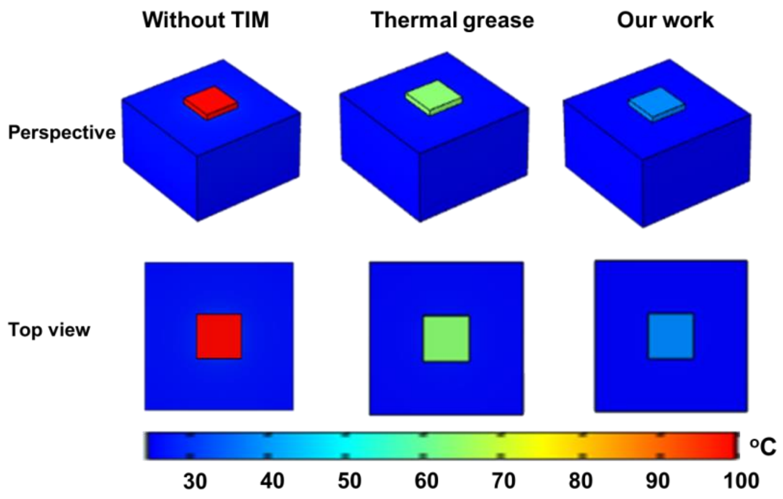

Fig. S9 The temperature distribution of the simulated system without TIM, using thermal grease and the polymer/s-BN composite film

S3 Supplementary Tables

Table S1 The data for crosslinking degree of the SA in the dried composite film*

Calcium ion concentration (wt%)	Film casting thickness(μm)	Freezing time (min)	Sample weight before soaked (g)	Sample weight after soaked (g)
0.5	200	10	0.1522	0.1044
0.8	200	10	0.1322	0.1320
3	200	10	0.1345	0.1345
5	200	10	0.1424	0.1424
3	400	10	0.1352	0.1352
3	600	10	0.1534	0.1534
3	800	10	0.1743	0.0825
3	200	1	0.1328	0.0943
3	200	3	0.1453	0.1453
3	200	6	0.1374	0.1374
3	200	8	0.1394	0.1393

* The crosslinking degree of the SA in the dried composite film is determined by comparing its weight change before and after complete soaking in water. If the weight of SA film obtained by ice-press assembly strategy didn't change before and after soaking in water for 24 h, it was completely cross-linked by calcium ions.

Table S2 The detailed information of Fig. 3b

Filler content	$K_{through\text{-}plane}$ ($Wm^{-1} K^{-1}$)	STC ($Wm^{-1} K^{-1}$/wt%)	Shape	Method	Refs.
27.05vol%	5.13	0.13	Bulk	wet-spinning	[S1]
21.9vol%	4.22	0.13	Bulk	Modified filtration	[S2]
18.2wt%	4.53	0.24	Bulk	Ice-template	[S3]
60vol%	6.11	0.07	Bulk	Sacrificial template	[S4]
8.35vol%	3.87	0.32	Bulk	Ice template	[S5]
4.4vol%	1.56	0.24	Bulk	Ice-template	[S6]
50wt%	9.8	0.20	Bulk	Freeze-drying	[S7]
53wt%	7.3	0.14	Bulk	CVD	[S8]
40wt%	3.85	0.096	Bulk	Hot-press	[S9]
60wt%	7.62	0.13	Bulk	Layer-by-layer stacking	[S10]
16wt%	1.15	0.07	Film	Tetrahedral skeleton template	[S11]
40wt%	7.29	0.18	Film	Vacuum filtration	[S12]
15wt%	9.6	0.64	Film	Ice-press assembly	[S13]
25wt%	21.3	0.85	Film	Ice-press assembly	Our work

Table S3 The detailed parameters of the components for simulated analysis

Components	Size (μm×μm)	Thermal conductivity (W m^{-1} K^{-1})
Filler	10×0.01	300(in-plane); 10(through-plane)
Polymer	100×20	0.18

Thin layers with a thickness of 1 nm are added on the interfaces between the filler and the polymer, and the thermal resistances value of the thin layer are set according to the different fillersThe heat sources are attached to the bottom surface of the composite and the other boundaries have an initial value at 20 °C. All the models are set as the same conditions and the heating time is 0.0006 s.

Table S4 The detailed parameters of the components in the simulated cooling system

Components	Size (cm×cm×cm)	Materials	Thermal conductivity (W m^{-1} K^{-1})	Specific heat capacity (J g^{-1} K^{-1})
CPU heater	3×3×0.1	Silicon	130	0.7
TIM	3×3×0.08	/	/	/
Heat sink	6×6×3	Aluminum alloy	238	0.9

This system simulates the heat transfer process from a CPU (54 W) to the heat sink kept at 25 °C.The steady-state temperature profiles of the simulated system are calculated according to the setting TIMs.

Supplementary References

[S1] C. Lei, Y. Zhang, D. Liu, X. Xu, K. Wu et al., Highly thermo-conductive yet electrically insulating material with perpendicularly engineered assembly of boron nitride nanosheets. Compos. Sci. Technol. **214**, 108995 (2021). https://doi.org/10.1016/j.compscitech.2021.108995

[S2] C. Xiao, Y. Guo, Y. Tang, J. Ding, X. Zhang et al., Epoxy composite with significantly improved thermal conductivity by constructing a vertically aligned three-dimensional network of silicon carbide nanowires/ boron nitride nanosheets. Compos. Part B Eng. **187**, 107855 (2020). https://doi.org/10.1016/j.compositesb.2020.107855

[S3] M.A. Kashfipour, R. Dent, N. Mehra, X. Yang, J. Gu et al., Directional xylitol crystal propagation in oriented micro-channels of boron nitride aerogel for isotropic heat conduction. Compos. Sci. Technol. **182**, 107715 (2019). https://doi.org/10.1016/j.compscitech.2019.107715

[S4] X. Xu, R. Hu, M. Chen, J. Dong, B. Xiao et al., 3D boron nitride foam filled epoxy composites with significantly enhanced thermal conductivity by a facial and scalable approach. Chem. Eng. J. **397**, 125447 (2020). https://doi.org/10.1016/j.cej.2020.125447

[S5] Y. Yao, Z. Ye, F. Huang, X. Zeng, T. Zhang et al., Achieving significant thermal conductivity enhancement via an ice-templated and sintered BN-SiC skeleton. ACS Appl. Mater. Interfaces **12**(2), 2892-2902 (2020). https://doi.org/10.1021/acsami.9b19280

[S6] X. Wang, P. Wu, 3D vertically aligned BNNS network with long-range continuous channels for achieving a highly thermally conductive composite. ACS Appl Mater. Interfaces **11**(32), 28943-28952 (2019). https://doi.org/10.1021/acsami.9b09398

[S7] J. Wang, D. Liu, Q. Li, C. Chen, Z. Chen et al., Lightweight, Superelastic yet thermoconductive boron nitride nanocomposite aerogel for thermal energy regulation. ACS Nano **13**(7), 7860-7870 (2019). https://doi.org/10.1021/acsnano.9b02182

[S8] Y. Xue, X. Zhou, T. Zhan, B. Jiang, Q. Guo et al., Densely interconnected porous bn frameworks for multifunctional and isotropically thermoconductive polymer composites. Adv. Funct. Mater. **28**(29), 1801205 (2018). https://doi.org/10.1002/adfm.201801205

[S9] X. Li, C. Li, X. Zhang, Y. Jiang, L. Xia et al., Simultaneously enhanced thermal conductivity and mechanical properties of PP/BN composites via constructing reinforced segregated structure with a

trace amount of BN wrapped PP fiber. Chem. Eng. J. **390**, 124563 (2020). https://doi.org/10.1016/j.cej.2020.124563

[S10] Q. Hu, X. Bai, C. Zhang, X. Zeng, Z. Huang et al., Oriented BN/Silicone rubber composite thermal interface materials with high out-of-plane thermal conductivity and flexibility. Compos. Part A Appl. Sci. Manuf. **152**, 106681 (2022). https://doi.org/10.1016/j.compositesa.2021.106681

[S11] H. Hong, Y.H. Jung, J.S. Lee, C. Jeong, J.U. Kim et al., Anisotropic thermal conductive composite by the guided assembly of boron nitride nanosheets for flexible and stretchable electronics. Adv. Funct. Mater. **29**(37), 1902575 (2019). https://doi.org/10.1002/adfm.201902575

[S12] L. An, R. Gu, B. Zhong, J. Wang, J. Zet al., Quasi-isotropically thermal conductive, highly transparent, insulating and super-flexible polymer films achieved by cross linked 2D hexagonal boron nitride nanosheets. Small **17**(46), 2101409 (2021). https://doi.org/10.1002/smll.202101409

[S13] T. Huang, F. Yang, T. Wang, J. Wang, Y. Li et al., Ladder-structured boron nitride nanosheet skeleton in flexible polymer films for superior thermal conductivity. Appl. Mater. Today **26**, 101299 (2022). https://doi.org/10.1016/j.apmt.2021.101299
